# Impact of metabolism and growth phase on the hydrogen isotopic composition of microbial fatty acids

**DOI:** 10.3389/fmicb.2015.00408

**Published:** 2015-05-08

**Authors:** Sandra M. Heinzelmann, Laura Villanueva, Danielle Sinke-Schoen, Jaap S. Sinninghe Damsté, Stefan Schouten, Marcel T. J. van der Meer

**Affiliations:** ^1^Department of Marine Organic Biogeochemistry, NIOZ Royal Netherlands Institute for Sea ResearchDen Burg, Netherlands; ^2^Department of Earth Sciences, Faculty of Geosciences, Utrecht UniversityUtrecht, Netherlands

**Keywords:** metabolism, fatty acids, hydrogen isotopic fractionation, growth phase

## Abstract

Microorganisms are involved in all elemental cycles and therefore it is important to study their metabolism in the natural environment. A recent technique to investigate this is the hydrogen isotopic composition of microbial fatty acids, i.e., heterotrophic microorganisms produce fatty acids enriched in deuterium (D) while photoautotrophic and chemoautotrophic microorganisms produce fatty acids depleted in D compared to the water in the culture medium (growth water). However, the impact of factors other than metabolism have not been investigated. Here, we evaluate the impact of growth phase compared to metabolism on the hydrogen isotopic composition of fatty acids of different environmentally relevant microorganisms with heterotrophic, photoautotrophic and chemoautotrophic metabolisms. Fatty acids produced by heterotrophs are enriched in D compared to growth water with ε_lipid/water_ between 82 and 359‰ when grown on glucose or acetate, respectively. Photoautotrophs (ε_lipid/water_ between −149 and −264‰) and chemoautotrophs (ε_lipid/water_ between −217 and −275‰) produce fatty acids depleted in D. Fatty acids become, in general, enriched by between 4 and 46‰ with growth phase which is minor compared to the influence of metabolisms. Therefore, the D/H ratio of fatty acids is a promising tool to investigate community metabolisms in nature.

## Introduction

Microorganisms are key players in all elemental cycles and therefore have a huge impact on their immediate and the global environment (Conrad, 1996; Morel and Price, 2003; Arrigo, 2005; Falkowski and Godfrey, 2008; Muyzer and Stams, 2008; Hügler and Sievert, 2011; Orcutt et al., 2011). In order to comprehend their environmental impact, it is important to characterize and understand their metabolic activities. Several approaches help to understand microbial metabolisms present in different environments. One approach is the isolation or enrichment of microorganisms from a specific environment to test its growth on possible substrates and investigate its metabolic pathways. Unfortunately, the isolation of specific microorganisms can give a biased view of the composition of microbial communities as it has been estimated that only ~1% of all microorganisms can be enriched, isolated, and cultivated by standard techniques (Amann et al., 1995). Often microorganisms with new metabolic capacities or that are present in the highest abundance have not been isolated (Overmann, 2006). Therefore, studying microbial activity *in situ* becomes necessary in order to understand metabolic dynamics within microbial communities.

For this purpose e.g., stable isotope probing (SIP) can be used to identify specific microorganisms which utilize particular substrates (Nold and Ward, 1996; Radajewski et al., 2000). The specific substrates have to be highly enriched in a stable isotope (e.g., D, ^13^C, ^15^N, ^18^O) for the label to be incorporated by active microorganisms into biomarkers like DNA, RNA and lipids. The labeled biomarkers can be then purified and identified (Boschker et al., 1998; Manefield et al., 2002; Radajewski et al., 2003; Dumont and Murrell, 2005; van der Meer et al., 2005, 2007; Neufeld et al., 2007). The most common approach to characterize the metabolic activity of microbial communities is estimate activity rate measurements of a specific activity (Chapelle and Lovley, 1990; Phelps et al., 1994). An alternative to this is the characterization of functional genes which are involved in different metabolic pathways using messenger RNA (mRNA) and 16S ribosomal RNA (Holmes et al., 2005). This approach allows not only for the identification of members of the community by gene sequence but also their relative abundance by determination of the copy number of that sequence and their metabolic activity by mRNA copy numbers (Corredor et al., 2004; Henry et al., 2004; Holmes et al., 2005; Sharma et al., 2007; Jensen et al., 2008; Agrawal and Lal, 2009; Blazejak and Schippers, 2011; Kong et al., 2012; Akerman et al., 2013). However, all the approaches listed above have their limitations like isotopic cross-labeling, artificial change in both microbial diversity and activity as a result of experiment set-up of incubations, or requires pre-knowledge of gene sequences (Radajewski et al., 2000; Dumont and Murrell, 2005; van der Meer et al., 2005; Cebron et al., 2007; Bowen et al., 2014). An alternative is to use the natural isotopic composition of lipids. For example, carbon isotope discrimination (δ^13^C) can be used for identification of methanotrophs due to the fact that they produce lipids depleted in ^13^C compared to other microorganisms (Summons et al., 1994).

Recently it has been shown that the ratio of deuterium to hydrogen (D/H or δD) of fatty acids reflect the central metabolism of microorganisms (Zhang et al., 2009a). Microbes grown under phototrophic conditions produce fatty acids depleted in D (ranging from −150 to −250‰) relative to the growth medium under both oxic and anoxic conditions (Sessions et al., 1999; Chikaraishi et al., 2004; Zhang and Sachs, 2007; Zhang et al., 2009a). Fatty acids of chemoautotrophs are even more depleted in D (ranging from −250 to −400‰) relative to the growth medium, independent of the electron donor (Valentine et al., 2004; Campbell et al., 2009; Zhang et al., 2009a). In contrast, organisms grown under heterotrophic conditions, e.g., grown with acetate or glucose as substrate, are relatively enriched in D and range from −150 to > +200‰ regardless of factors such as temperature (Sessions et al., 2002; Zhang et al., 2009a; Dirghangi and Pagani, 2013; Fang et al., 2014). Zhang et al. (2009a) attributed these differences to the D/H ratio of nicotinamide adenine dinucleotide phosphate (NADPH), which is generated by a variety of different reactions in different metabolic pathways (each associated with different hydrogen isotopic fractionations) and subsequently used as the main H source in lipid biosynthesis (Saito et al., 1980; Robins et al., 2003; Schmidt et al., 2003). The analysis of the D-composition of microbial fatty acids may thus yield insights into the metabolism of individual microbes or microbial communities. Furthermore, the persistence of lipids over geological time periods should allow for the study of microbial metabolisms in the past from sedimentary records. However, not many microbes have yet been analyzed for the hydrogen isotopic composition of fatty acids. Furthermore, other factors than metabolism have been shown to influence the D/H ratio of lipids such as temperature (Zhang et al., 2009b; Dirghangi and Pagani, 2013), lipid biosynthetic pathways (Fang et al., 2014), growth rate, growth phase, and salinity (Schouten et al., 2006; Wolhowe et al., 2009; Chivall et al., 2014; M'boule et al., 2014).

In order to improve the reliability of δD of fatty acids as an indicator for the metabolism of microorganisms we evaluated both the effect of metabolism (auto- vs. heterotrophic) and of growth phase (exponential, stationary, and death phase) on the δD-values of fatty acids of different microorganisms which are mainly derived from aquatic environments with salinities ranging from almost freshwater or open marine. *Thiocapsa roseopersicina* and *Halochromatium glycolicum* are both anaerobic, phototrophic purple sulfur bacteria using hydrogen sulfide as electron donor and found in microbial mat and saline lakes, respectively. *Isochrysis galbana* is an aerobic, phototrophic haptophyte algae using water as electron donor and common in coastal marine environments. *Thiobacillus denitrificans* is an anaerobic, chemolithoautotrophic β-*proteobacterium* using thiosulfate as electron donor and is common in aquatic environment from freshwater to marine. Finally, a recently isolated *Pseudomonas* str. LFY10 from Lake Fryxell, Dry Valleys, Antarctica, is investigated which is an aerobic, heterotrophic γ-*proteobacterium* using either glucose or acetate as carbon source.

## Materials and methods

### Cultures

The photoautotrophic purple sulfur bacteria *Thiocapsa roseopersicina* (DSM-217) and *Halochromatium glycolicum* (DSM-11080) were grown on a modified Pfenning's medium containing 0.34 g NH_4_Cl, 0.34 g KH_2_PO_4_, 0.5 g MgSO_4_ × 7 H_2_O, 0.34 g KCl, 0.25 g CaCl_2_ × 2 H_2_O, 1.5 g NaHCO_3_, 0.4 g Na_2_S × 9 H_2_O, 0.02 g vitamin B_12_, and 1 mL trace element solution SL-12 (Pfennig, 1965) per liter of distilled water. The pH was adjusted with 1 M HCl to pH 7-7.5. The medium for *H. glycolicum* was additionally supplemented with 6% NaCl, 0.3% MgCl_2_ × 6 H_2_O, and 0.05% Na_2_S_2_O_3_(final concentration). The cultures were incubated in air tight bottles at 25°C and a light intensity of ~1300 lux of a halogen lamp (16 h light, 8 h dark).

The chemolithoautotrophic sulfide oxidizer *Thiobacillus denitrificans* (DSM-12475) was grown on a medium containing 2 g KH_2_PO_4_, 2 g KNO_3_, 1 g NH_4_Cl, 0.8 g MgSO_4_ × 7 H_2_O, 2 mL trace element solution SL-4, 5 g Na_2_S_2_O_3_ × 7 H_2_O, 1 g NaHCO_3_, 2 mg FeSO_4_ × 7 H_2_O, 1 mL 0.1 N H_2_SO_4_ per liter of distilled water (pH 7.0). The trace element solution SL-4 contained 0.5 g EDTA, 0.2 g FeSO_4_ × 7 H_2_O, 0.01 g ZnSO_4_ × 7 H_2_O, 3 mg MnCl_2_ × 4 H_2_O, 0.03 g H_3_BO_3_, 0.02 g CoCl_2_ × 6 H_2_O, 1 mg CuCl_2_ × 2 H_2_O, 2 mg NiCl_2_ × 6 H_2_O, 3 mg Na_2_MoO_4_ × 2 H_2_O per liter of distilled water. *T. denitrificans* cultures were incubated at 25°C.

The photoautotrophic eukaryote *Isochrysis galbana* (CCMP 1323) was grown at a salinity of 35.5 practical salinity units (psu) as previously described (M'boule et al., 2014) in f/2 medium which contained 0.07 g of NaNO_3_, 0.013 g of Na_2_HPO_4_ × 12 H_2_O, 1 mL of a trace element solution, and 1 mL of a vitamin solution per 1 L of sea water (Guillard, 1975). The trace element solution contained per liter of distilled water: 4.36 g EDTA, 3.15 g FeCl_3_ × 6 H_2_O, 0.01 g CuSO_4_ × 5 H_2_O, 0.02 g ZnSO_4_ × 7 H_2_O, 0.01 g CoCl_2_ × 6 H_2_O, 0.1 g MnCl_2_ × 4 H_2_O, and 4.8 mg Na_2_MoO_4_ × 2 H_2_O. The vitamin solution contained per liter of distilled water: 0.5 mg biotin, 0.1 g vitamin B_1_, and 0.5 mg vitamin B_12_. The cultures were incubated at 15°C and a light intensity of ~3000 lux of a cool white fluorescent light (16 h light, 8 h dark).

A recently isolated heterotrophic *Pseudomonas* str. LFY10 obtained from Prof. Matt Sattley (Indiana Wesleyan University, Marion, IN) was grown on an ammonium–glucose medium and an ammonium–acetate medium. The ammonium–glucose medium contained: 5 g glucose, 0.2 g MgSO_4_ × 7 H_2_O, 5 g NaCl, 1.3 g (NH_4_)_2_HPO_4_, 1 g KH_2_PO_4_, 2 mL trace element solution SL-4 per liter of distilled water (pH 7.1). The ammonium–acetate medium contained 5 g Na–acetate, 0.2 g MgSO_4_ × 7 H_2_O, 5 g NaCl, 1.3 g (NH_4_)_2_HPO_4_, 1 g KH_2_PO_4_, 2 mL trace element solution SL-4 per liter of distilled water. The pH was adjusted to 7.1. The cultures were incubated at 25°C.

After inoculation cell densities, and thereby growth phase, were monitored regularly by flow cytometry (BD Accuri™ C6, San Jose USA) or by measuring the optical density (OD) at 600 nm with a spectrometer (Molecular Devices SpectraMax M2, Sunnyvale USA). Culture samples, including water samples for hydrogen isotope analysis, were taken during exponential, stationary and death phase. The water was stored with no headspace in 12 mL exertainers (Labco) in the dark at ~5°C until analysis. Biomass was collected by filtration over a 0.7 μm GF/F filter (Whatman, GE Healthcare Life Sciences, Little Chalfont, UK) or by centrifugation.

### Lipid extraction

Bacterial biomass and filters were freeze dried and hydrolyzed directly by base hydrolysis with four volumes of 1 N KOH in methanol (MeOH) solution under reflux for 1 h at 190°C. Afterwards the pH was adjusted to 4 with 2 N HCl/MeOH (1/1) and the liquid was transferred into a separatory funnel. The residues were further extracted once with MeOH/H_2_O (1/1), twice with MeOH, and three times with dichloromethane (DCM) (two volumes each). The extracts were combined and bidistilled H_2_O (six volumes) was added. The combined solutions were mixed and allowed to separate in a MeOH/H_2_O and DCM phase, the DCM phase was removed and collected. The MeOH/H_2_O layer was re-extracted twice with 3 mL DCM. The combined DCM layers were dried over a Na_2_SO_4_ column and the DCM was evaporated under a stream of nitrogen. The dried extracts were stored at 4°C before further workup.

Fatty acids were methylated with a boron trifluoride–methanol solution (BF_3_–MeOH) for 5 min at 60°C. Then H_2_O and DCM were added (1 mL each). The aqueous layer was washed three times with 1 mL DCM, and the combined DCM fractions were cleaned over a Na_2_SO_4_ column and dried under a stream of nitrogen. In order to obtain a fatty acid fraction, the methylated extract was separated over an aluminum oxide (AlOx) column, eluting the methylated fatty acids with DCM.

In order to identify the position of double bonds in unsaturated fatty acids, the methylated fatty acids were derivatized with dimethyldisulfide (DMDS) (Nichols et al., 1986). Hexane, DMDS and I_2_/ether (60 mg/mL) were added to the fatty acids and incubated at 40°C overnight. After adding hexane, the iodine was deactivated by addition of a 5% aqueous solution of Na_2_S_2_O_3_. The aqueous phase was washed twice with hexane. The combined hexane layers were cleaned over a Na_2_SO_4_ column and dried under a stream of nitrogen. The dried extracts were stored at 4°C before analysis.

### Fatty acid analysis

The fatty acid fractions were analyzed by gas chromatography (GC) using an Agilent 6890 gas chromatograph with a flame ionization detector (FID) using a fused silica capillary column (25 m × 320 μm) coated with CP Sil-5 (film thickness 0.12 μm) with helium as carrier gas. The temperature program was the following: initial temperature 70°C, increase of temperature to 130°C with 20°C min^−1^, and then to 320°C with 4°C min^−1^ for 10 min. Individual compounds were identified by GC/mass spectrometry (GC/MS) using a Agilent 7890A GC instrument and Agilent 5975C VL mass selective detector (MSD).

### Hydrogen isotope analysis

Hydrogen isotope analysis of the fatty acids was performed by GC thermal conversion isotope ratio monitoring MS (GC/TC/irMS) using an Agilent 7890 GC connected via Thermo GC Isolink and Conflo IV interfaces to a Thermo Delta V MS according to Chivall et al. (2014). Samples were injected onto an Agilent CP-Sil 5 CB column (25 m × 0.32 mm ID; 0.4 μm film thickness; He carrier gas, 1.0 mL min^−1^). The GC temperature program was 70 to 145°C at 20°C min^−1^, then to 200°C at 4°C min^−1^ 320°C for 15 min. Eluting compounds were converted to H_2_ at 1420°C in a ceramic tube before introduction to the mass spectrometer. An internal standard, squalane (δ D = −170‰), was co-injected with each fatty acid sample in order to monitor the precision of the measurements. The average δD of the internal standard was −1.7 ± 3‰. The δD of the individual fatty acids was measured in duplicates and corrected for the added methyl group.

The hydrogen isotopic composition of fatty acids compared to water was expressed as ε_lipid/water_ following:

$$\varepsilon =\left(\frac{1000+\delta {\text{D}}_{\text{FA}}}{1000+\delta {\text{D}}_{\text{water}}}-1\right)*1000$$

The δD of the water was determined by injecting at least 10 × 1 μL on an elemental analysis/thermal conversion/isotope ratio monitoring MS (EA/TC/irMS) using a Thermo Finnigan TC/EA interfaced via a Thermo Finnigan ConFlo III to a Thermo Finnigan Delta^+^ XL mass spectrometer following the procedure described by M'boule et al. (2014) with North Sea water (δD = 5‰) and bidistilled water (δD = −76‰) as standards.

The δD-value of dry sodium acetate was also determined by EA/TC/irMS. NBS 22 mineral oil (δD = −120.0‰) and polyethylene IAEA-CH-7 (δD = −100.3‰) were used as standards. The δD-value of acetate was measured in triplicate.

The hydrogen isotopic composition of the non-exchangeable hydrogen of the glucose substrate was determined by analyzing the acetylated derivative of glucose. For this, glucose was acetylated using 0.5 mL acetic anhydride with a pre-determined δD-value and 0.5 mL pyridine for 3 h at 75°C. Afterwards, 1 mL distilled H_2_O was added and the water layer was washed three times with hexane. The hexane was evaporated and the acetylated glucose was dissolved in ethyl acetate and analyzed by GC, GC–MS and GC/TC/irMS similar to the fatty acids. The δD-value of the non-exchangeable hydrogen of glucose was calculated by correcting for the added acetyl groups. The acetylated glucose was measured five times.

Statistical analysis was done via One-Way ANOVA test with SigmaPlot Version 12.0 (Systat Software, Inc., San Jose, USA).

## Results

### Thiocapsa roseopersicina

**Figure 1:**
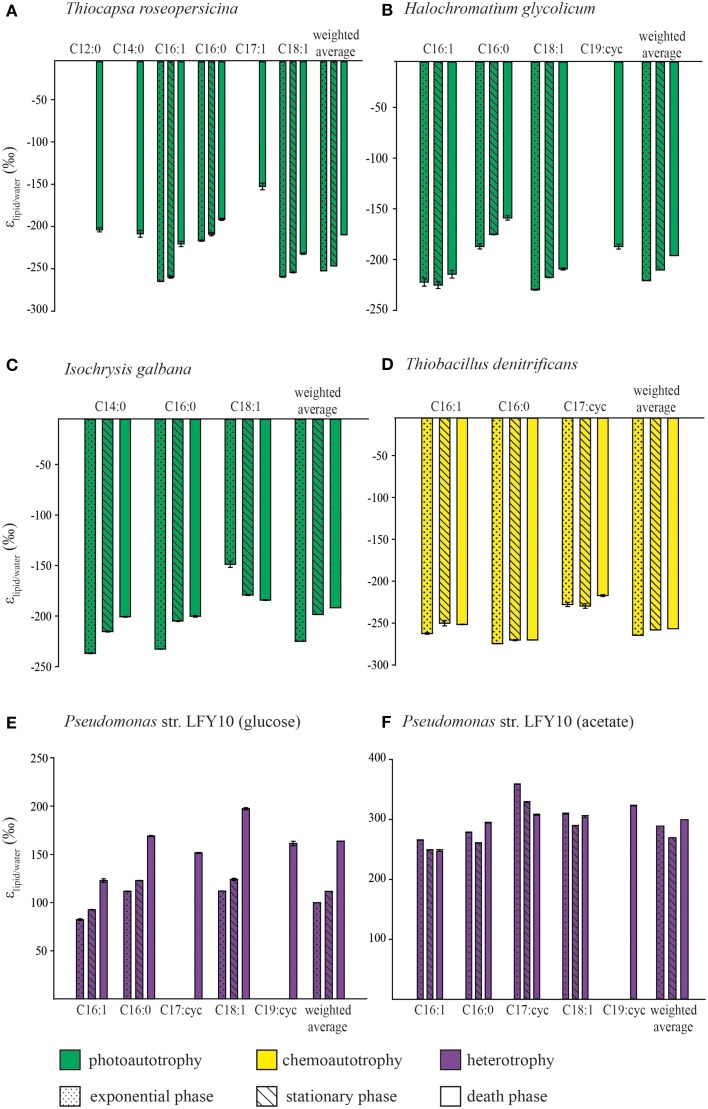
**The D/H fractionation between fatty acids and culture medium observed in different growth phases during the culture experiments**. Plotted are the mean ε-values (lipid vs. water). Error bars are the standard deviation of the duplicate measurements of the fatty acids. **(A)** *Thiocapsa roseopersicina*, **(B)** *Halochromatium glycolicum*, **(C)** *Isochrysis galbana*, **(D)** *Thiobacillus denitrificans* and *Pseudomonas* str. LFY10 grown on **(E)** glucose and **(F)** acetate. Also plotted is the weighted average ε_lipid/water_ of the fatty acids.

*Thiocapsa roseopersicina* was grown photoautotrophically under anoxic conditions with CO_2_as sole carbon source and hydrogen sulfide as electron donor. Fatty acids that were present in all growth phases are C16:1ω 7, C16:0, and C18:1ω 7. In addition, during the death phase traces of C12:0, C14:0, and C17:1ω 7 were also detected. The C18:1 fatty acid (30-49%) was the most abundant fatty acid in all growth phases followed by C16:1 (21-32%) and C16:0 (~21%). Minor fatty acids were C12:0, C14:0, and C17:1 fatty acids with abundances between 7 and 14% (Table S1). All fatty acids were depleted in D relative to the growth medium (all δD-values are summarized in Table S2) and the hydrogen isotopic fractionation expressed as ε_lipid/water_ between the fatty acids and the growth water of the individual fatty acids ranged between −153 and −264‰ (Table 1). Fatty acids were most depleted during exponential growth and most enriched during the death phase (Figure 1A, Table 1).

**Table 1:** **D/H fractionation between fatty acids and growth medium for fatty acids produced by different microorganisms under various metabolic conditions**.

**Organism**	**Substrate**	**δD_substrate_ (‰)**	**δD_water_ (‰)**	**Mean ε_lipid/water_ (‰)**								**Weighted. av. (‰)**	**GP**
				**C12:0**	**C14:0**	C16:1^*^	**C16:0**	**C17:cyc**	**C17:1ϕ**	**C18:1Φ**	**C19:cyc**		
*Thiocapsa*	CO_2_,	–	−51 ± 3			−264	−216			−259		−252	E
*roseopersicina*	light		−50 ± 3			−260	−209			−254		−247	S
			−59 ± 3	−204	−209	−221	−191		−153	−232		−210	D
*Halochromatium*	CO_2_,	–	−50 ± 2			−222	−187			−230		−221	E
*glycolicum*	light		−51±2			−225	−175			−218		−210	S
			−61±2			−214	−159			−209	−187	−196	D
*Isochrysis*	CO_2_,	–	4±2		−237		−233			−149		−225	E
*galbana*	light		5±2		−215		−205			−179		−198	S
			9±1		−201		−200			−184		−192	D
*Thiobacillus*	CO_2_	–	−51 ± 1			−262	−275	−228				−265	E
*denitrificans*			−49 ± 3			−250	−270	−229				−258	S
			−54 ± 3			−252	−270	−217				−257	D
*Pseudomonas*	glucose	−8 ± 11	−56 ± 2			82	111			112		100	E
str. LFY10			−55 ± 2			93	123			124		112	S
			−38 ± 2			123	169	152		197	161	164	D
*Pseudomonas*	acetate	−1.8 ± 3	−57 ± 2			265	278	359		309		289	E
str. LFY10			−57 ± 3			249	261	328		290		270	S
			−44 ± 2			247	294	307		304	323	300	D

C16:1^*^, double bond at the ω 7 position; C17:1ϕ, double bond at the ω 7 position; C18:1ϕ, double bond in all cultures except for I. galbana (ω 9) at the ω 7 position; GP, growth phase; E, exponential; S, stationary; D, death.

### Halochromatium glycolicum

*Halochromatium glycolicum* was grown photoautotrophically, under anoxic conditions with CO_2_as sole carbon source and hydrogen sulfide as electron donor. The fatty acids C16:1ω 7, C16:0, and C18:1ω 7 were present in all growth phases with C18:1 fatty acid being the most abundant (52-73%) and C16:1 the least abundant (7-9%) (Table S1). In addition, a C19 fatty acid containing a cyclopropane moiety (C19:cyc) was only present in the death phase. All measured fatty acids were depleted in D relative to the growth medium (Table S2) and ε_lipid/water_ for the individual fatty acids ranged between −159 and −230‰ (Table 1). The fatty acids were most depleted during exponential growth and most enriched during the death phase (Figure 1B, Table 1). The C16:0 fatty acid was enriched in D by 40-50‰ compared to the other fatty acids in all growth phases.

### Isochrysis galbana

*Isochrysis galbana* was grown photoautotrophically under oxic conditions with CO_2_as sole carbon source (M'boule et al., 2014). In all growth phases, C14:0, C16:0, and C18:1ω 9 fatty acids were identified as well as various other unsaturated C18 and a polyunsaturated C22 fatty acids. Either C14:0 or C18:1 fatty acids are in general the most abundant fatty acids (Table S1). All fatty acids were depleted in D compared to the growth medium (Table S2) and ε_lipid/water_-values ranged from −149 to −237‰ (Table 1). The C14:0 and C16:0 fatty acids were most depleted in D during exponential growth and became enriched by up to 30‰ with increasing age of the culture. On the other hand C18:1ω 9 fatty acid was most enriched during exponential growth and became depleted in D with age of the culture by up to 35‰ (Figure 1C; Table 1). The D/H ratio of the other unsaturated C18 and a polyunsaturated C22 fatty acids could not be measured with certainty due to either incomplete separation or low abundance.

### Thiobacillus denitrificans

*Thiobacillus denitrificans* was grown chemoautotrophically with thiosulfate as electron donor. In all growth phases C16:1ω 7 and C16:0 fatty acids were present in equal abundance. Minor amounts of C17:cyc were also detected (Table S1). All fatty acids were depleted in D relative to the growth medium (Table S2) with ε_lipid/water_ of the individual fatty acids ranging between −217 and −275‰ (Table 1). In general, fatty acids were most depleted during exponential growth (Figure 1D). The C16:0 fatty acid was depleted by 10-50‰ compared to the other fatty acids in all growth phases.

### *Pseudomonas str*. LFY10

The *Pseudomonas* sp. strain was grown heterotrophically on either glucose or acetate as carbon source under oxic conditions. On both substrates, *Pseudomonas* str. LFY10 produced C16:1ω 7, C16:0, and C18:1ω 7 FA. The C17:cyc fatty acids was identified in all growth phases when grown on acetate but only during the death phase when grown on glucose. In the death phase, C19:cyc fatty acid was identified when grown on either substrate (Table S1). There were no differences in fatty acid distribution between exponential and stationary phase with C16:1 and C16:0 being the most abundant fatty acids when grown on glucose and C16:0 being the most abundant fatty acid when grown on acetate (Table S1). In the culture grown on glucose all fatty acids during death phase were enriched in D relative to the growth medium (Table S2) with ε_lipid/water_ ranging between 82 and 197‰ (Table 1), as well as to the substrate, with ε_lipid/substrate_ ranging between 30 and 161‰ (Table 2). All fatty acids became enriched with age of culture (Figure 1D). In the cultures grown on acetate, all fatty acids were significantly enriched compared to both the growth medium and the substrate. The ε_lipid/water_-value of the individual fatty acids ranged between 247 and 359‰ (Table 1), while the ε_lipid/substrate_ of the individual fatty acids was between 351 and 469‰ (Table 2). Fatty acids of *Pseudomonas* str. LFY10 grown on acetate did not show a general enrichment in D with progressing growth phase (Figure 1F).

**Table 2:** **D/H fractionation between fatty acids and growth substrate for fatty acids produced by *Pseudomonas* str. LFY10**.

**Substrate**	**δD_water_ (‰)**	**δD_substrate_ (‰)**	**ε_lipid/water_ (‰)**					**GP**
			C16:1^*^	**C16:0**	**C17:cyc**	**C18:1Φ**	**C19:cyc**	
Glucose	−56 ± 2	−8 ± 11	30	58		58		E
	−55 ± 2		40	69		70		S
	−38 ± 2		89	134	117	161	126	D
Acetate	−57 ± 2	−1.8 ± 3	369	383	469	416		E
	−57 ± 3		351	363	436	394		S
	−44 ± 2		366	418	432	429	450	D

C16:1^*^, double bond at the ω 7 position; C18:1ϕ, double bond at the ω 7 position. GP, growth phase; E, exponential, S, stationary, D, death.

## Discussion

### Influence of metabolism on the δd of C16:0 fatty acid

**Figure 2:**
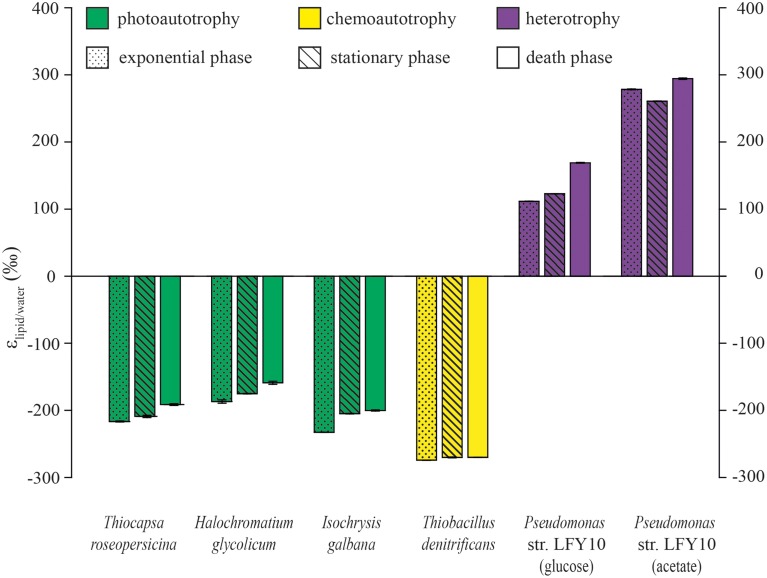
**Impact of growth phase (exponential, stationary and death phase) on the hydrogen isotopic fractionation of the C16:0 fatty acid of the different cultivated microbes**. Plotted are the mean ε-values of the duplicate measurements of the fatty acids and error bars are the standard deviation of the duplicate measurements of the fatty acids. Cultures are *Thiocapsa roseopersicina*, *Halochromatium glycolicum*, *Isochrysis galbana*, *Thiobacillus denitrificans* and *Pseudomonas* str. LFY10 grown on glucose and acetate.

The C16:0 fatty acid is the most common and often most abundant fatty acid in bacterial and eukaryotic microorganisms (Gunstone et al., 2012). Indeed, all the cultures tested in this study synthesize at least the C16:0 fatty acid, while other fatty acids were often either absent or present in lower amounts. Therefore, for comparing the hydrogen isotopic fractionation of the different microbes we focus on the C16:0 fatty acid (Figure 2) and discuss the patterns in more detail below. Furthermore, the weighted average of all measured fatty acids for each culture showed the same trend as the C16:0 fatty acid indicating that changes in the hydrogen isotopic composition of individual fatty acids is not strongly affected by the relative abundance of the fatty acids (Table 1, Figure 1).

#### Photoautotrophs

The hydrogen isotopic fractionation for the C16:0 fatty acid (ε_C16:0/water_) of all photoautotrophs, *Thiocapsa roseopersicina*, *Halochromatium glycolicum*, and *Isochrysis galbana*, is relatively similar and ranged from −187 to −233‰. The oxygenic photoautotroph *I. galbana* reduce NADP^+^ to NADPH using H_2_O as electron donor (Lengeler et al., 1999) and thus the sole source of hydrogen is water. The D/H ratio of fatty acids depends mainly on the fractionation associated with the splitting of water, the reduction of NADP^+^ to NADPH, the transfer of H to the initial photosynthate and the transfer of H during fatty acid biosynthesis (Hayes, 2001). The anoxygenic photoautotrophs *Thiocapsa roseopersicina* and *Halochromatium glycolicum* both reduce NADP^+^ via reverse electron transport (Imhoff, 2006). Depending on the pH, H_2_S is soluble in water and forms S^2−^ and 2 H^+^ with the latter being exchangeable with the protons of water. Since the amount of H_2_S is relatively small compared to water (0.04%), the D/H ratio of water will not be substantially affected. The similar fractionation of anoxygenic and oxygenic photoautotrophs suggest that the steps leading to the production of NADPH under both conditions have similar fractionation factors. The same has been observed for various other algae and an anoxygenic, photoautotrophic bacterium (Zhang and Sachs, 2007; Zhang et al., 2009a).

#### Chemolithoautotrophs

The C16:0 fatty acid of the chemolithoautotrophic *T. denitrificans* is more depleted in D compared to those of the photoautotrophs (Figure 2) even though water is also the most likely source for hydrogen here. *T. denitrificans* contains an electron transport chain to reduce NAD^+^ to NADH by quinone–cytochrome b (Beller et al., 2006). The electron transport chain, the enzymes involved and the substrate reduced are different from photoautotrophic microorganisms, which could potentially explain the negative offset in fatty acid D/H ratios. Another possibility could be that *T. denitrificans* might, like *T. thioparus* to which it is physiologically similar (Kelly and Wood, 2000), rather use NADH than NADPH as hydrogen source during fatty acid biosynthesis. Matin and Rittenberg (1971) also suggested that in obligate chemolithoautotrophic *Thiobacilli* NADH, and not NADPH, is used for reducing power during biosynthesis. Additionally, at least some heterotrophs have been shown to contain a NADH–NADPH converting transhydrogenase. In case of excess NADPH the enzyme leads to reduction of NAD^+^ to NADH while oxidizing NADPH to NADP^+^. This would leave the remaining NADPH pool enriched and the NADH strongly depleted in D (Zhang et al., 2009a). This could be an additional fractionation effect that potentially contributes to the relatively depleted fatty acids in chemoautotrophs which use NADH. However, the genome of *Thiobacillus denitrificans* does not contain a NADH–NADPH converting transhydrogenase (Beller et al., 2006), although we cannot exclude the possibility it contains enzymes with a similar function.

#### Heterotrophs

The heterotroph *Pseudomonas* str. LFY10 produces D-enriched fatty acids compared to D-depleted fatty acids in all autotrophs. Furthermore, similar to *Escherichia coli* (Zhang et al., 2009a), ε_lipid/water_ of the individual fatty acids of *Pseudomonas* str. LFY10 is higher, when grown on acetate than on glucose. In heterotrophic organisms an important hydrogen source is the organic substrate used as carbon and energy source. All *Pseudomonas* species possess the tricarboxylic acid (TCA) cycle, the pentose phosphate pathway (Moore et al., 2006) and possibly also a NADH–NADPH converting transhydrogenase (Louie and Kaplan, 1970; French et al., 1997). The observed enrichment in D is suggested to occur during NADP^+^ reduction in the TCA cycle or due to conversion of excess NADPH to NADH via the NADH–NADPH converting transhydrogenase (Zhang et al., 2009a). Some of the supplied acetate might also be used as a direct building block during fatty acid biosynthesis and therefore some of the H of fatty acids would come directly from acetate. When *Pseudomonas* sp. is grown on glucose, NADP^+^ will be reduced in the pentose phosphate pathway in addition to the TCA cycle. While NADP^+^ reduction in the TCA cycle leads to NADPH enriched in D, the reduction in the pentose phosphate pathway might lead to more depleted NADPH. The mixed NADPH pool could thus explain why the fatty acids produced by *Pseudomonas* str. LFY10 are less enriched in D when grown on glucose compared to acetate.

### Influence of growth phase on the δd of fatty acids

In addition to metabolism we investigated the effect of growth phase. Fatty acids produced by the different microorganisms are in general, but not exclusively, increasingly enriched in D with increasing age of the culture. From exponential to stationary phase, the ε_lipid/water_-values of the C16:0 fatty acids of all but one culture increase by around 10‰. Only for *Pseudomonas* str. LFY10 grown on acetate a depletion of 15‰ was observed (Figure 2).

Few studies have focused on the impact of growth phase on D/H ratios of lipids. Interestingly, Wolhowe et al. (2009) and Chivall et al. (2014) showed that alkenones produced by haptophyte algae are more depleted in D in stationary growth phase compared to exponential growth phase, which is in contrast to the enrichment observed here for the C16:0 fatty acid as well as the weighted average of fatty acids in *I. galbana* (Figure 1C). This is interesting considering that the C16:0 fatty acid is assumed to serve as a precursor for the synthesis of alkenones (Rontani et al., 2006; Wallace, 2012). A possible explanation is that the C16:0 is produced mainly in the chloroplast, while it is assumed that the alkenones are produced in the cytosol by chain elongation (Wallace, 2012). Therefore, two different NADPH pools could be used for the biosynthesis of alkenones and C16:0 fatty acid, respectively. The observed isotopic difference with growth phase could be due to a decrease in structural lipid synthesis such as fatty acids since the algae are no longer growing and dividing as a result of nutrient limitation. At the same time NADPH is still produced during photosynthesis leading to a surplus of reducing power. This excess NADPH will then be used for the production of storage products, such as alkenones, which do not contain limiting elements like N or P (Wolhowe et al., 2009). Alternatively, when a smaller fraction of the relatively D depleted fatty acids are used for structural components such as membranes, they may be used for alkenone biosynthesis resulting in more D depleted alkenones with growth phase.

Zhang et al. (2009a) already reported that heterotrophic microorganisms like *Cupriavidus necator* and *C. oxalaticus* produce fatty acids during exponential growth that are more enriched in D compared to fatty acids produced during stationary phase (ε_lipid/water_ 169 vs. 70‰ and 149 vs. 95‰, respectively) when grown on succinate. Like acetate, succinate is metabolized via the TCA cycle which may thus lead to production of NADPH enriched in D. Thus, for heterotrophs, growth on substrates that are directly involved in the TCA cycle apparently leads to a depletion in D of all fatty acids, including C16:0, when shifting from exponential to stationary phase. In contrast, growth on substrates that are involved in the pentose phosphate pathway, like glucose, apparently leads to enrichment in D of fatty acids in stationary phase compared to exponential phase.

Although growth phase changes result in changes in D/H ratios of fatty acids these are relatively minor compared to the impact of metabolism on fatty acid D/H ratios. Therefore, changes in growth phase in microbial communities in the environment can be considered to have relatively minor impact on the overall isotopic signal of the fatty acid pool of the whole community. Additionally, lipid identity does not play a major role on the hydrogen isotopic composition of fatty acids compared to metabolism. The ε_lipid/water_-values of the different fatty acids with the same initial biosynthetic pathways fall in a similar range and differences observed are due to the addition and/or removal of hydrogen atoms related with chain length and degree of unsaturation.

### Application of δd of fatty acids as a population metabolism indicator

**Figure 3:**
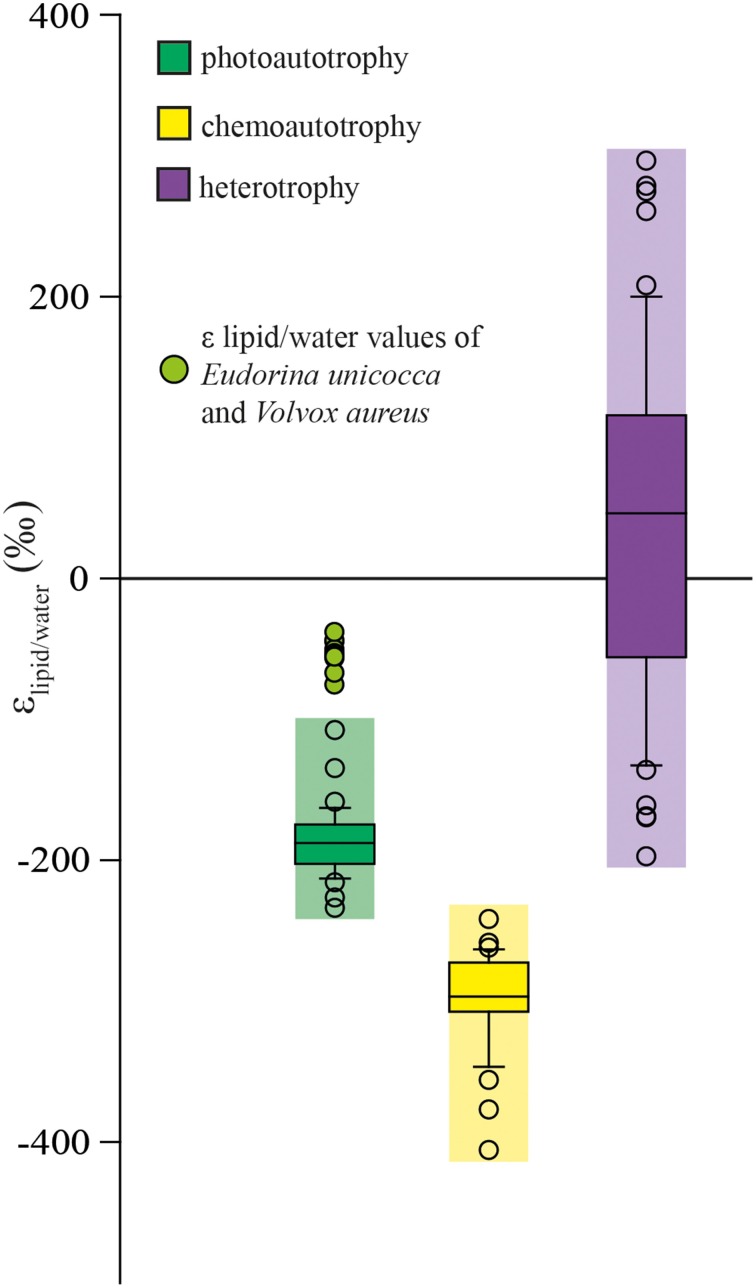
**Box plots of D/H fractionations between the C16:0 fatty acid and culture medium observed in different culture experiments**. Cultures included from this study are *Thiocapsa roseopersicina*, *Halochromatium glycolicum*, *Isochrysis galbana*, *Thiobacillus denitrificans*, and *Pseudomonas* str. LFY10. Additionally, published data for *Isochrysis galbana*, *Ascophyllum* sp., *Alexandrium fundyense*, *Methylococcus capsulatus*, *Saragassum filicinum*, *Undararia pinnatifida*, *Binghamia californica*, *Gelidium japonica*, *Sporomusa* sp., *Botrycoccus braunii*, *Eudorina unicocca*, *Volvox aureus*, *Desulfobacterium autotrophicum*, *Cupriavidus oxalaticus*, *Cupriavidus necator*, *Escherichia coli*, *Rhodopseudomonas palustris*, *Tetrahymena thermophile*, and *Moritella japonica* DSK 1 have been included (Sessions et al., 1999, 2002; Chikaraishi et al., 2004; Valentine et al., 2004; Zhang and Sachs, 2007; Campbell et al., 2009; Zhang et al., 2009a; Dirghangi and Pagani, 2013; Fang et al., 2014).

When we summarize all published (including this study) ε_C16:0/water_-values of different microorganisms grown as either photoautotroph, chemoautotroph or heterotroph (Sessions et al., 1999, 2002; Chikaraishi et al., 2004; Valentine et al., 2004; Zhang and Sachs, 2007; Campbell et al., 2009; Zhang et al., 2009a; Dirghangi and Pagani, 2013; Fang et al., 2014), the three metabolism types show distinct, but slightly overlapping ranges (Figure 3). Microorganisms grown as photoautotrophs produce fatty acids which are depleted in D relative to the growth medium with the majority of ε_C16:0/water_-values ranging around −170 to −200‰. The only exceptions are the cultures of the freshwater algae *Eudorina unicocca* and *Volvox aureus* which produce D-enriched fatty acids compared to other photoautotrophs (Zhang and Sachs, 2007). A possible explanation could be that both *Eudorina unicocca* and *Volvox aureus* are colony forming algae which, unlike the colony forming algae *Botryococcus braunii* (Zhang and Sachs, 2007), consist of two different cell types, somatic cells and reproductive (gonidia) cells (Herron et al., 2009). Possible differences in the metabolism between these two different cell types could play a role in the relatively enrichment in D of the C16:0 fatty acid compared to other photoautotrophs. Due to the fact that the ε_lipid/water_-values of these two organisms do not seem to follow the pattern observed for the C16:0 fatty acids produced by all photoautotrophs we have indicated them separately in our summary in Figure 3. Thus, C16:0 fatty acid of photoautotrophs have a mean ε_C16:0/water_-value of −186‰ and range between −162 and −215‰ (95% confidence interval, *n* = 34). Chemoautotrophically grown microorganisms produce fatty acids with a mean ε_C16:0/water_-value of −298‰ and ranging between −264 and −345‰ (95% confidence interval, *n* = 32) which is depleted by ca. 100‰ relative to fatty acids produced by photoautotrophs. Heterotrophically grown microorganisms typically show an enrichment in D of the lipids relative to the water (with a mean ε_C16:0/water_-value of 39‰ and ranging between −133 and 199‰ (95% confidence interval, *n* = 53). The differences between the different metabolisms are significant (*P* < 0.001) and should allow for the characterization of the dominant metabolism of microbial communities in the environment by analyzing the isotopic difference between C16:0 fatty acid and water. However, several issues should be kept in mind. Relatively few microorganisms have been analyzed for their fatty acid hydrogen isotopic composition, and although most of them fit the general pattern it is possible that exceptions to this pattern arise once other microorganisms are analyzed in the future. In the environment all microorganisms producing the C16:0 fatty acid will contribute to the fatty acid pool in varying amounts depending on the amount produced in the cell. Therefore, the ε_C16:0/water_-value in an environmental sample will not so much represent the average ε_lipid/water_-value of the whole microbial community but will also be affected by the relative abundance of the C16:0 fatty acid in the various contributing microbes. For instance, the pink streamer (PS) communities in Yellowstone National Park are dominated by chemoautotrophic *Aquificales* which mainly produce C20:1, C21:cyc with C18:0 and C16:0 fatty acids occurring only in minor amounts, while C16:0 fatty acid is abundant in members of the co-occurring genus *Thermus* which are heterotrophic. Thus, the D/H ratio of the C16:0 fatty acid reflects heterotrophy rather than chemoautotrophy in these PS communities despite the dominance of chemoautotrophs (Osburn et al., 2011).

Nevertheless, in order to obtain an idea of the dominating metabolism of a microbial community in the present, as well as in the past, the ε_C16:0/water_-value is a promising approach, possibly combined with the ε_lipid/water_-value of other fatty acids and potentially biomarker lipids with a more restricted origin.

## Conclusion

The hydrogen isotopic composition of fatty acids produced by a range of different microorganisms depends on the general metabolism expressed during growth. Both photoautotrophs and chemoautotrophs produce fatty acids strongly depleted in D, while heterotrophs produce fatty acids enriched in D compared to the growth medium. Fatty acids produced during different growth phases become somewhat enriched in D with increasing age of the culture in most of the experiments described here. Thus, growth phase likely plays a minor role in controlling the D/H ratio of fatty acids relative to metabolism in the natural environment. Our results suggest that an overall characterization of community metabolism via the D/H ratio of fatty acids is potentially feasible.

### Conflict of interest statement

The authors declare that the research was conducted in the absence of any commercial or financial relationships that could be construed as a potential conflict of interest.
