# Supplementary material for: Impact of metabolism and growth phase on the hydrogen isotopic composition of microbial fatty acids
Source: Front Microbiol. 2015 May 8;6:408. doi: 10.3389/fmicb.2015.00408 (PMC4424904; doi:10.3389/fmicb.2015.00408)
Supplement: Supplementary file 1 [file Table1.DOCX]

**Supplementary data**:

**Table S1**

Fatty acid abundance in different microorganisms grown under various metabolic conditions.

| Organism | Fatty acid abundance [%] | | | | | | | | GP |
| --- | --- | --- | --- | --- | --- | --- | --- | --- | --- |
|  | C12:0 | C14:0 | C16:1* | C16:0 | C17:cyc | C17:1ᵠ | C18:1ᶲ | C19:cyc |  |
| *Thiocapsa roseopersicina* | - | - | 32.4 | 20.0 | - | - | 47.7 | - | E |
|  | - | - | 30.5 | 20.7 | - | - | 48.8 | - | S |
|  | 14.3 | 7.3 | 20.9 | 20.5 | - | 7.4 | 29.6 | - | D |
| *Halochromatium glycolicum* | - | - | 6.9 | 19.8 | - | - | 73.3 | - | E |
|  | - | - | 8.4 | 18.9 | - | - | 72.7 | - | S |
|  | - | - | 9.3 | 18.4 | - | - | 52.4 | 19.9 | D |
| *Isochrysis galbana* | - | 55.5 | - | 32.5 | - | - | 12.0 | - | E |
|  | - | 36.6 | - | 23.4 | - | - | 39.9 | - | S |
|  | - | 23.6 | - | 23.0 | - | - | 53.4 | - | D |
| *Thiobacillus denitrificans* | - | - | 47.2 | 43.6 | 9.2 | - | - | - | E |
|  | - | - | 47.3 | 46.2 | 6.6 | - | - | - | S |
|  | - | - | 46.6 | 44.3 | 9.1 | - | - | - | D |
| *Pseudomonas* str. LFY10  (glucose) | - | - | 39.8 | 35.0 | - | - | 25.2 | - | E |
|  | - | - | 38.3 | 35.9 | - | - | 25.8 | - | S |
|  | - | - | 6.9 | 47.8 | 25.1 | - | 10.6 | 9.6 | D |
| *Pseudomonas* str. LFY10  (acetate) | - | - | 29.2 | 44.4 | 12.3 | - | 14.1 | - | E |
|  | - | - | 29.1 | 44.6 | 11.9 | - | 14.3 | - | S |
|  | - | - | 4.7 | 40.4 | 39.9 | - | 9.2 | 5.7 | D |

C16:1*: double bond at the ω7 position; C17:1ᵠ: double bond at the ω7 position; C18:1ᶲ: double bond in all cultures except for *I. galbana* (ω9) at the ω7 position.; GP: growth phase; E= exponential, S= stationary, D= death
