# Supplementary material for: Impact of metabolism and growth phase on the hydrogen isotopic composition of microbial fatty acids
Source: Front Microbiol. 2015 May 8;6:408. doi: 10.3389/fmicb.2015.00408 (PMC4424904; doi:10.3389/fmicb.2015.00408)
Supplement: Supplementary file 2 [file Table2.DOCX]

**Table S2**

Mean δD values of fatty acids produced by different microorganisms under various metabolic conditions.

| Organism | growth rate | δD_water_  ‰ | mean δD ‰ | | | | | | | | GP῀ |
| --- | --- | --- | --- | --- | --- | --- | --- | --- | --- | --- | --- |
|  |  |  | C12:0 | C14:0 | C16:1* | C16:0 | C17:cyc | C17:1ᵠ | C18:1ᶲ | C19:cyc |  |
| *Thiocapsa roseopersicina* | 0.11 | -51±3 |  |  | -302 | -256 |  |  | -297 |  | E |
|  | n.d. | -50±3 |  |  | -297 | -249 |  |  | -291 |  | S |
|  | n.d. | -59±3 | -251 | -255 | -267 | -239 |  | -203 | -277 |  | D |
| *Halochromatium glycolicum* | 0.12 | -50±2 |  |  | -261 | -228 |  |  | -268 |  | E |
|  | n.d. | -51±2 |  |  | -264 | -217 |  |  | -257 |  | S |
|  | n.d. | -61±2 |  |  | -262 | -210 |  |  | -257 | -237 | D |
| *Isochrysis galbana* | 0.62ᵠ | 4±2 |  | -234 |  | -229 |  |  | -145 |  | E |
|  | n.d. | 5±2 |  | -211 |  | -201 |  |  | -175 |  | S |
|  | n.d. | 9±1 |  | -193 |  | -193 |  |  | -177 |  | D |
| *Thiobacillus denitrificans* | 0.93 | -51±1 |  |  | -300 | -312 | -267 |  |  |  | E |
|  | n.d. | -49±3 |  |  | -287 | -306 | -267 |  |  |  | S |
|  | n.d. | -54±3 |  |  | -292 | -309 | -259 |  |  |  | D |
| *Pseudomonas* str. LFY10  (glucose) | n.d. | -56±2 |  |  | 22 | 50 |  |  | 50 |  | E |
|  | n.d. | -55±2 |  |  | 32 | 61 |  |  | 62 |  | S |
|  | n.d. | -38±2 |  |  | 81 | 125 | 108 |  | 152 | 118 | D |
| *Pseudomonas* str. LFY10  (acetate) | n.d. | -57±2 |  |  | 194 | 206 | 282 |  | 235 |  | E |
|  | n.d. | -57±3 |  |  | 179 | 189 | 252 |  | 216 |  | S |
|  | n.d. | -44±2 |  |  | 192 | 237 | 250 |  | 247 | 265 | D |

C16:1*: double bond at the ω7 position; C17:1ᵠ: double bond at the ω7 position; C18:1ᶲ: double bond in all cultures except for *I. galbana* (ω9) at the ω7 position.; GP: growth phase; E= exponential, S= stationary, D= death; ᵠ value previously reported by Chivall et al. 2014
